# Optimizing an Injectable Composite Oxygen-Generating System for Relieving Tissue Hypoxia

**DOI:** 10.3389/fbioe.2020.00511

**Published:** 2020-05-26

**Authors:** Tai-En Hsieh, Sheng-Ju Lin, Li-Chi Chen, Chun-Chieh Chen, Po-Liang Lai, Chieh-Cheng Huang

**Affiliations:** ^1^Institute of Biomedical Engineering, National Tsing Hua University, Hsinchu, Taiwan; ^2^Department of Life Science, National Tsing Hua University, Hsinchu, Taiwan; ^3^Department of Orthopaedic Surgery, Linkou Chang Gung Memorial Hospital, Taoyuan City, Taiwan; ^4^Bone and Joint Research Center, Linkou Chang Gung Memorial Hospital, Taoyuan City, Taiwan

**Keywords:** hypoxia, hypoxia-inducible factor, oxygen-generating biomaterials, peroxide, PLGA microparticles

## Abstract

Oxygen deficiency resulting from bone fracture-induced vascular disruption leads to massive cell death and delayed osteoblast differentiation, ultimately impairing new bone formation and fracture healing. Enhancing local tissue oxygenation can help promote bone regeneration. In this work, an injectable composite oxygen-generating system consisting of calcium peroxide (CaO_2_)/manganese dioxide (MnO_2_)-encapsulated poly lactic-*co*-glycolic acid (PLGA) microparticles (CaO_2_ + MnO_2_@PLGA MPs) is proposed for the local delivery of oxygen. By utilizing a series of methodologies, the impacts of each component used for MP fabrication on the oxygen release behavior and cytotoxicity of the CaO_2_ + MnO_2_@ PLGA MPs are thoroughly investigated. Our analytical data obtained from *in vitro* studies indicate that the optimized CaO_2_ + MnO_2_@PLGA MPs developed in this study can effectively relieve the hypoxia of preosteoblast MC3T3-E1 cells that are grown under low oxygen tension and promote their osteogenic differentiation, thus holding great promise in enhancing fractural healing by increasing tissue oxygenation.

## Introduction

Bone fracture is one of the most common injuries of the musculoskeletal system (Claes et al., 2012). Although bone tissue possesses excellent self-healing capability, it has been reported that ~10% of patients with bone fracture exhibit healing problems, such as delayed union or non-union (Zeitouni et al., 2012; Wang et al., 2017). As oxygen is required in several basic cellular processes that are crucial for fracture healing (Lu et al., 2013), tissue hypoxia resulting from vascular disruption during fracture leads to cell death and delayed osteoblast differentiation, ultimately impairing new bone formation and fracture healing (Lu et al., 2013; Gómez-Barrena et al., 2015).

To address this issue, autologous bone grafts can be harvested and transplanted to the defect site to promote bone regeneration owing to their endogenous high-density vasculature, which can quickly anastomose with surrounding blood vessels (Zheng et al., 2010; Lee and Volpicelli, 2017). However, limited donor tissue availability and severe additional post-operative pain and complications restrict clinical applications of this approach (Ho et al., 2016). Other strategies, such as hyperbaric oxygen (HBO) therapy, have been employed to increase tissue oxygenation (Lu et al., 2013; Park and Park, 2018). As oxygen is still delivered via the vasculature, however, it is unlikely that poorly perfused injury tissue would benefit greatly from HBO treatment (Farris et al., 2016). Additionally, sustained oxygen delivery cannot be achieved by using HBO therapy, which can only be applied in a transient manner to avoid unwanted side effects (Farris et al., 2016). Hence, a sustained oxygen delivery strategy that can be used to improve local oxygen tension is warranted.

Several peroxide-based oxygen-evolving materials, such as calcium peroxide (CaO_2_) (Wang J. et al., 2011; Steg et al., 2015; Huang et al., 2016) and sodium percarbonate (Harrison et al., 2007; Ward et al., 2013; Chandra et al., 2015), have been utilized to chemically generate oxygen to enhance tissue oxygenation. Upon exposure to water, peroxide materials break down to yield hydrogen peroxide (H_2_O_2_), which can be further converted into molecular oxygen and water by catalysts (Farris et al., 2016; Gholipourmalekabadi et al., 2016). As the rate of oxygen generation depends on several environmental factors (Gholipourmalekabadi et al., 2016), such as humidity, pH, temperature, and the presence of catalysts, peroxide materials have been incorporated in a variety of biomaterials in the formats of films (Harrison et al., 2007), particles (Newland et al., 2018), and fibers (Wang J. et al., 2011) to achieve the sustained release of oxygen. This technique has been explored to provide oxygen for application in the field of regenerative medicine, including ischemic diseases (Harrison et al., 2007), cell transplantation (Pedraza et al., 2012; Coronel et al., 2017; Fan et al., 2018), wound healing (Chandra et al., 2015), and tissue engineering (Oh et al., 2009).

Although several peroxide-based oxygen delivery vehicles have been developed, some common limitations, especially the initial burst release of oxygen occurring in the first few hours and the production of cytotoxic H_2_O_2_ or other reactive oxygen species, remain major challenges (Farris et al., 2016; Gholipourmalekabadi et al., 2016; Park and Park, 2018). Even though the precise balance oxygen delivery and cytotoxicity can be overcome by utilizing various material-based strategies (Steg et al., 2015), most of these methods result in significantly increased size of the developed oxygen-delivery platform, thus reducing its feasibility in clinical application (Pedraza et al., 2012). Moreover, the capacity of these oxygen-generating carriers to promote osteogenic differentiation under low oxygen tension has not yet been investigated.

**Figure 1:**
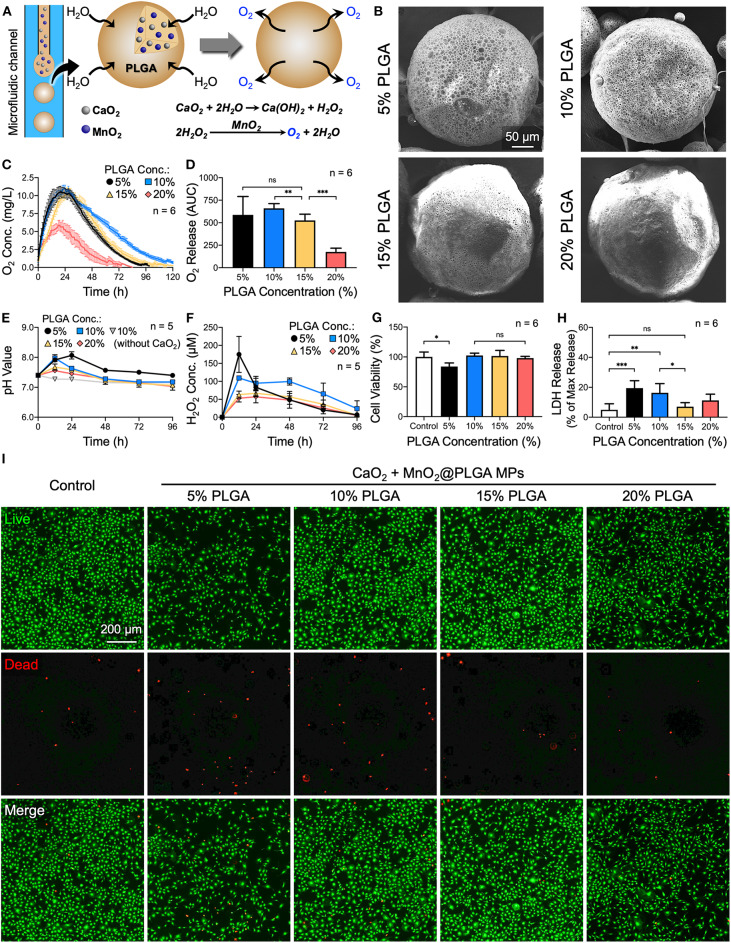
Effects of PLGA concentration on the oxygen release behavior of CaO_2_ + MnO_2_@PLGA MPs. **(A)** Schematic diagram showing the structure and composition of the developed CaO_2_ + MnO_2_@PLGA MPs that act as an oxygen-generating system to enhance tissue oxygenation. **(B)** SEM images of CaO_2_ + MnO_2_@PLGA MPs prepared using various PLGA concentrations. **(C)** The *in vitro* release profiles and **(D)** the corresponding AUCs of oxygen release from CaO_2_ + MnO_2_@PLGA MPs incubated in PBS at 37°C. **(E)** Variation in pH and **(F)** accumulation of H_2_O_2_ in test saline following treatment with various MPs. **(G)** Results of the CCK-8 assay, **(H)** LDH assay, and **(I)** live/dead staining following treatment with various MPs. *p < 0.05; **p < 0.01; ***p < 0.005; ns, not significant.

To address the abovementioned issues, we present an innovative implantable composite oxygen-generating system that can be easily delivered by local injection, and characterize its function in relieving cellular hypoxia and promoting osteogenic differentiation (Figure 1A). To fabricate a composite oxygen-generating system, CaO_2_ and manganese dioxide (MnO_2_) (Peng et al., 2017) were used as a source of H_2_O_2_ and a catalyst, respectively. In aqueous environments, H_2_O_2_ is first formed and then decomposes into oxygen. In addition to oxygen generation, the calcium ions (Ca^2+^) produced during CaO_2_ hydrolysis may be utilized as a calcium source for new bone formation (Teotia et al., 2016). To prolong the duration of oxygen evolution, CaO_2_ and MnO_2_ powders were embedded into poly(lactic-*co*-glycolic acid) (PLGA) microparticles (MPs; CaO_2_ + MnO_2_@PLGA MPs) using a microfluidic device (Figure 1A). To achieve the precise balance between oxygen generation and the caused cytotoxicity, a multifactor experimental design was employed, resulting in a more comprehensive understanding of the fashions in which oxygen evolves and the byproducts are generated from the MPs during the reaction. This study lays the solid foundation for the future development of more sophisticated and clinically applicable oxygen-generating biomaterials. We hypothesize that the developed injectable oxygen-generating system can be employed to increase local oxygen tension and thus enhance the osteogenesis of preosteoblasts and their mineral deposition *in vitro*, thereby holding great promise in promoting new bone formation and fracture healing.

## Materials and Methods

### Materials

CaO_2_, MnO_2_, polyvinyl alcohol (PVA), ascorbic acid, β-glycerophosphate, Alizarin red S, and hexadecylpyridinium chloride monohydrate were purchased from Sigma-Aldrich (St. Louis, MO, USA). PLGA (with a lactide:glycolide molar ratio of 75:25) was acquired from Green Square Material (Taipei, Taiwan). The mouse preosteoblast MC3T3-E1 cells were purchased from the American Type Culture Collection (ATCC CRL-2593, Manassas, VA, USA), while cell culture reagents were procured from Thermo Fisher Scientific (Waltham, MA, USA). All other chemicals and reagents used were of analytical grade.

### CaO_2_ + MnO_2_@PLGA MP Preparation

An oil-in-water single-emulsion microfluidic system with poly(vinyl chloride) tubes, glass capillaries, and 23G needles was used to prepare the CaO_2_ + MnO_2_@PLGA MPs. The oil phase, a solution of PLGA in dichloromethane (DCM) that contained CaO_2_ and MnO_2_ powder, was introduced into the microfluidic system at a flow rate of 0.25 mL/min to form single-emulsion droplets in a water phase that contained 5 wt% PVA at a flow rate of 5 mL/min. The formed emulsified CaO_2_ + MnO_2_@PLGA MPs were added to a larger water phase, which was stirred for 4 hours, allowing the solvent to evaporate. The CaO_2_ + MnO_2_@PLGA MPs were then washed in deionized water to remove the outer PVA before being stored at 4°C for further use. The morphology of the prepared CaO_2_ + MnO_2_@PLGA MPs was examined under a scanning electron microscope (SEM; JSM-5600, JEOL, Tokyo, Japan). Additionally, the specific surface areas of the developed MPs were assessed by nitrogen adsorption using an ASAP 2020 analyzer (Micromeritics, Norcross, GA, USA).

### Oxygen Release Behavior of CaO_2_ + MnO_2_@PLGA MPs

The prepared CaO_2_ + MnO_2_@PLGA MPs were transferred into phosphate-buffered saline (PBS) and incubated in a hypoxia chamber with 1% oxygen at 37°C (ProOx 110, BioSpherix, Parish, NY, USA). The dissolved oxygen concentrations were monitored using an InLab OptiOx DO sensor (Mettler Toledo, Greifensee, Switzerland). The pH value and the concentration of accumulated H_2_O_2_ in PBS samples during oxygen evolution were evaluated using a pH meter (ST3100; OHAUS, Parsippany, NJ, USA) and the Amplex Red (Life Technologies) assay, respectively.

### Cytotoxicity of CaO_2_ + MnO_2_@PLGA MPs

Preosteoblast MC3T3-E1 cells were utilized to evaluate the cytotoxicity of CaO_2_ + MnO_2_@PLGA MPs. Cells were seeded at 7.5 × 10^3^ cells per well in 48-well plates that contained Minimum Essential Medium α (αMEM) supplemented with 10% fetal bovine serum (FBS; GE Healthcare Bio-Sciences, Pittsburgh, PA, USA) and incubated for 24 h. Subsequently, the prepared CaO_2_ + MnO_2_@PLGA MPs were employed for treating cells by direct transfer into each well. After incubation for another 24 h, the viability of the MC3T3-E1 cells was evaluated using a live/dead viability kit (Thermo Fisher Scientific), and the cells were photographed by a fluorescence microscope (Axio Observer 7; Carl Zeiss). Additionally, the cell viability and cytotoxicity were quantified using a Cell Counting Kit-8 (CCK-8; Dojindo Laboratories, Kumamoto, Japan) (Park et al., 2018) and a Cytotoxicity Lactate Dehydrogenase (LDH) Assay Kit (Dojindo Laboratories), respectively.

### Alleviation of Cellular Hypoxia by CaO_2_ + MnO_2_@PLGA MPs

To detect cellular hypoxia, MC3T3-E1 cells that were preincubated with Image-iT Green Hypoxia Reagent (Thermo Fisher Scientific) (Ayuso et al., 2016), a cell-permeable fluorogenic probe, were maintained in 1% oxygen (Heracell VIOS 160i incubator; Thermo Fisher Scientific) for 24 h. The cells were then treated with CaO_2_ + MnO_2_@PLGA MPs for an additional 24 h. Thereafter, the test cells were observed using a fluorescence microscope. Alternatively, test cells that were maintained under hypoxic conditions (1% oxygen) and treated with CaO_2_ + MnO_2_@PLGA MPs were fixed in 4% paraformaldehyde and then stained with a primary antibody against hypoxia-inducible factor (HIF)-1α (Abcam, Cambridge, MA, USA). After incubation with a secondary antibody and counterstaining with 4′,6-diamidino-2-phenylindole (Thermo Fisher Scientific), the samples were observed using a fluorescence microscope.

### Osteogenic Differentiation of MC3T3-E1 Cells

The effects of CaO_2_ + MnO_2_@PLGA MP treatment on the osteogenic differentiation of preosteoblast MC3T3-E1 cells under hypoxic conditions (1% oxygen) were investigated by evaluating the degree of *in vitro* mineralization. After incubating 7.5 × 10^3^ cells in each well of a 48-well plate for 24 h, the culture medium was replaced with αMEM supplemented with 10% FBS, 50 μg/mL ascorbic acid, 5 mM β-glycerophosphate, and test MPs. The cells were then cultivated in 1% oxygen for 20 days (Lin et al., 2018). The culture media together with the test MPs were replaced every 3 days throughout the entire experimental course. To assess the *in vitro* mineralization, cells were fixed in ice-cold 70% ethanol for 1 h and incubated with 40 mM Alizarin red S solution for 20 min. The stained calcium mineral deposition was observed under a microscope followed by dissolving in 10 wt% hexadecylpyridinium chloride solution and quantified by absorbance measurement at 562 nm using a microplate reader (SpectraMax iD3, Molecular Devices, Sunnyvale, CA, USA) (Sun et al., 2013).

### Animal Study

All animal experimental procedures were approved by the Institutional Animal Care & Utilization Committee, National Tsing Hua University, Hsinchu, Taiwan. Female BALB/c mice at 8 weeks of age were purchased from National Laboratory Animal Center, Nangang, Taiwan. The animals were anesthetized by inhalation of 2% isoflurane. After removing the hair of the dorsal side, the skin of the injection sites was sterilized using an alcohol swab. Test MPs (10 mg) were suspended in 200 μL normal saline and injected subcutaneously into the dorsal flanks of mice using a 23-gauge needle (*n* = 4). After 14 days, the mice were euthanized by CO_2_ inhalation. The dorsal tissue was harvested and embedded in optimum cutting temperature compound. Ten-micrometer thick tissue sections were cut using a cryostat microtome (CM1950, Leica, Wetzlar, Germany) and stained with hematoxylin and eosin (H&E). Photomicrographs of the stained sections were taken and processed for nuclei counting using ImageJ (Schneider et al., 2012). Briefly, five randomly selected views surrounding the implanted MPs were converted to greyscale and processed by the Watershed ImageJ plugin followed by cell nuclei counting using the Particle Analyzer tool.

### Statistical Analysis

All statistical analyses were performed using GraphPad Prism software (version 8.1; San Diego, CA, USA). The experimental data are presented as the mean ± standard deviation. A two-tailed Student's *t*-test was used for comparisons between two groups. One-way analysis of variance (ANOVA) with Bonferroni correction was used for comparisons of three or more groups. A *p* < 0.05 indicated a significant difference.

## Results and Discussion

### Concentration of PLGA Modulates Oxygen Release Behavior

In the first step of the reaction, CaO_2_ can produce both calcium hydroxide [Ca(OH)_2_] and H_2_O_2_ upon reaction with water. Therefore, the speed of water infiltration into MPs may determine the reaction rate as well as the duration of oxygen generation. In addition, the alteration of the pH environment caused by the produced Ca(OH)_2_ and accumulation of H_2_O_2_ may have potential harmful effects on the surrounding tissue (Gholipourmalekabadi et al., 2016). To maximize the oxygen-producing capability of MPs while minimizing their cytotoxic effects, the optimization of several parameters was performed.

As the polymeric matrix of PLGA can modulate the hydrolytic reaction of CaO_2_ by restricting the permeation of water molecules into MPs, we first investigated the effect of PLGA concentration on the morphology of MPs. As indicated by the SEM images in Figure 1B, the MPs that were prepared using 5% or 10% PLGA exhibited a porous surface architecture. In the 15 and 20% PLGA groups, however, less pores were observed. Determined using the Brunauer–Emmett–Teller method (Wang Y.-T. et al., 2011), the specific surface areas of the MPs (0.537 m^2^/g for 5% PLGA group, 0.406 m^2^/g for 10% PLGA group, 0.295 m^2^/g for 15% PLGA group, and 0.0855 m^2^/g for 20% PLGA group) further revealed their differences in porosity. During MP preparation, the emulsion was gently stirred in PVA solution for 4 h to remove the DCM by evaporation. Owing to the hydrophobicity of PLGA polymer, water may infiltrate more easily into MPs that were prepared using a low concentration of PLGA to initiate the reaction of oxygen production before complete solidification of the MPs, thus resulting in their porous morphology. In contrast, a high concentration of PLGA may act as a barrier to prevent the further penetration of water, thereby presenting a less porous architecture.

Next, to investigate their oxygen evolution profile, test MPs were suspended in PBS and incubated in a hypoxia chamber with 1% oxygen at 37°C. As indicated by the obtained oxygen release profiles (Figure 1C) and their corresponding areas under the curve (AUCs; the total area under the dissolved oxygen concentration vs. time curve; Figure 1D), the duration and amount of oxygen production were effectively modulated by the concentration of PLGA used to prepare MPs, which all encapsulated the same weight of CaO_2_ (7.5 mg/mL). The MPs prepared using 10% PLGA exhibited a longer period (~120 h) of oxygen generation than those fabricated using 5% PLGA (96 h). As the concentration of PLGA further increased up to 20%, the period and amount of oxygen production decreased significantly owing to the reduced surface areas of MPs (Figure 1B) and the hydrophobic characteristics of PLGA, which prevent water infiltration.

The impacts of MPs on the surrounding environment during oxygen evolution and the resultant cytotoxicity were also assessed. In the 5 and 10% PLGA groups, the relatively fast reaction led to dramatic changes in pH values (Figure 1E) and a significant accumulation of H_2_O_2_ (Figure 1F); both processes had an adverse effect on the viability of MC3T3-E1 cells, as indicated by the experimental results obtained from the CCK-8 assay, LDH assay, and live/dead staining (Figures 1G–I, respectively). As a result, MPs prepared using 15% PLGA that could evolve oxygen for ~100 h and had only limited cytotoxicity were chosen for the following experiments.

### Amounts of Encapsulated CaO_2_ and MnO_2_ Control Oxygen Release Behavior

**Figure 2:**
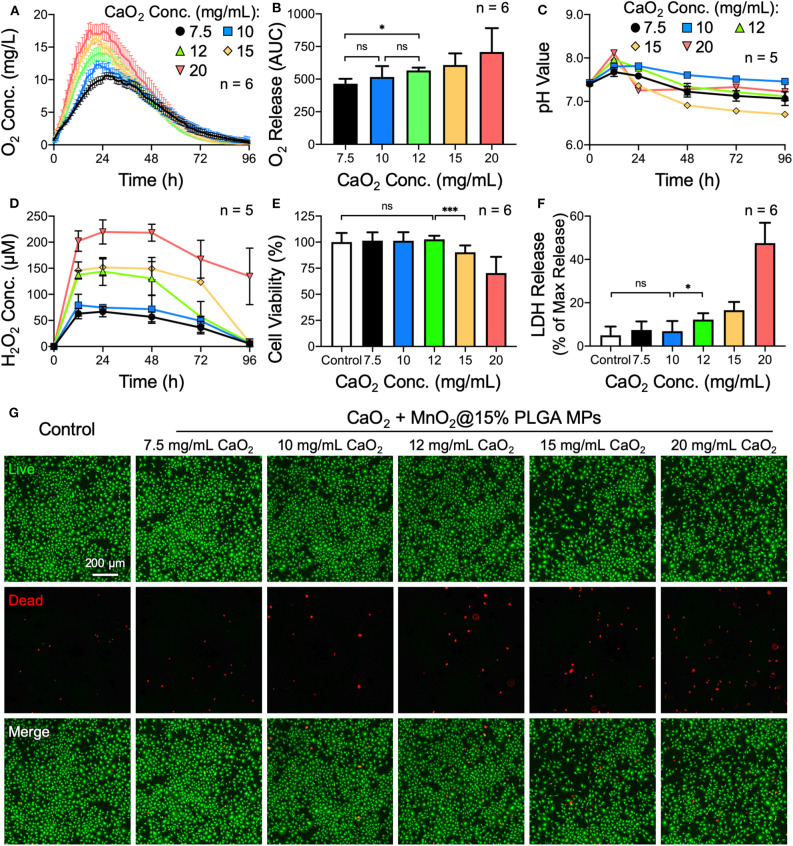
Effects of the amount of encapsulated CaO_2_ on the oxygen release behavior of CaO_2_ + MnO_2_@PLGA MPs. **(A)** The release profiles and **(B)** corresponding AUCs of oxygen release from MPs. **(C)** Variation in pH and **(D)** accumulation of H_2_O_2_ in PBS samples following treatment with MPs. **(E)** Results of the CCK-8 assay, **(F)** LDH assay, and **(G)** live/dead staining following treatment with MPs. *p < 0.05; ***p < 0.005; ns, not significant.

Next, the amount of CaO_2_ loaded in the MPs was optimized. As the CaO_2_ powder was used as the source of H_2_O_2_, which could be further converted to oxygen, we speculated that the more CaO_2_ was encapsulated, the more oxygen could be generated by the MPs. According to the oxygen generation profiles and their corresponding AUCs (Figures 2A,B), the total amount of oxygen evolved from the MPs elevated gradually as the amount of incorporated CaO_2_ increased, while the duration of oxygen generation was similar among all test groups. Additionally, the magnitude of alkalization and H_2_O_2_ accumulation in the PBS samples was positively correlated with the amount of encapsulated CaO_2_ (Figures 2C,D).

The MPs that were loaded with various amounts of CaO_2_ were then used to treat MC3T3-E1 cells. A significant cytotoxic effect was observed when 12 mg/mL or more CaO_2_ powder was encapsulated in the MPs (Figures 2E–G). Using the same methodology, the optimized amount of the catalyst MnO_2_ embedded in the MPs was determined to be 0.22 mg/mL (Supplementary Figure 1). Therefore, CaO_2_ + MnO_2_@PLGA MPs that were fabricated using 15% PLGA and loaded with 10 mg/mL CaO_2_ and 0.22 mg/mL MnO_2_ were employed for subsequent studies.

### CaO_2_ + MnO_2_@PLGA MPs Relieve Cellular Hypoxia

**Figure 3:**
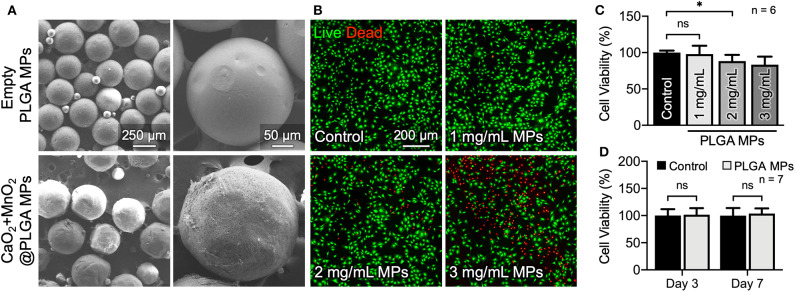
*In vitro* cytotoxicity evaluation of the developed CaO_2_ + MnO_2_@PLGA MPs. **(A)** SEM images of the PLGA MPs. **(B)** Representative live/dead images and **(C)** corresponding results of the CCK-8 assay at 24 h following treatment with various doses of MPs. **(D)** Results of CCK-8 assay of MC3T3-E1 cells receiving MP treatment for 3 or 7 days. *p < 0.05; ns, not significant.

The morphology and size of the as-prepared oxygen-generating MPs were examined by SEM. Figure 3A shows that the plain PLGA MPs were spherical in shape and had a diameter of 248 ± 19 μm (*n* = 5 batches). After encapsulating CaO_2_ and MnO_2_, however, the size of the PLGA MPs increased slightly (298 ± 62 μm in diameter). Before evaluating their capacity to alleviate cellular hypoxia, we first examined the optimal dose of CaO_2_ + MnO_2_@PLGA MPs for treating MC3T3-E1 cells. According to the results of live/dead staining (Figure 3B) and CCK-8 assay (Figure 3C), a dose of 1 mg/mL MPs did not exhibit significant cytotoxicity. To further confirm the safety of the determined dosage, the viability of the MC3T3-E1 cells that received 1 mg/mL MPs and were incubated for 3 and 7 days was evaluated. In this study, both the culture medium and the MPs were replaced every other day. During the entire culture period, the cells that were treated with the MPs exhibited a similar level of viability when compared to the untreated control (Figure 3D and Supplementary Figure 2), demonstrating the low cytotoxicity of the developed CaO_2_ + MnO_2_@PLGA MPs; thus, this dose was used in the subsequent reoxygenation and osteogenic differentiation studies.

**Figure 4:**
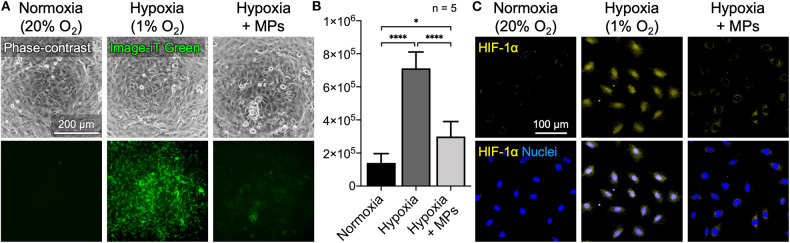
Alleviation of cellular hypoxia by CaO_2_ + MnO_2_@PLGA MPs. **(A,B)** Treating cells that were incubated under hypoxic conditions with the developed CaO_2_ + MnO_2_@PLGA MPs effectively relieved cellular hypoxia, as indicated by the reduced fluorescence intensity of hypoxia-sensing dye Image-iT Green and **(C)** the decreased nuclear translocation/accumulation of the key hypoxia-responsive transcription factor HIF-1α. *p < 0.05; ****p < 0.001.

The availability of oxygen for cells that were incubated under low oxygen tension (1% O_2_) with or without CaO_2_ + MnO_2_@PLGA MPs was assessed using the hypoxia-responsive dye Image-iT Green Hypoxia Reagent, whose fluorescent signal increases with reduced oxygen levels (Stone et al., 2019). Cells that were maintained under normoxic conditions (21% O_2_) were used as a control. For cells cultivated under normal oxygen tension, only a basal level of fluorescent signal could be detected (Figures 4A,B). Conversely, the cells incubated under hypoxic conditions emitted strong green fluorescence, demonstrating the limited availability of oxygen for cells. Moreover, the hypoxic cells that were treated with CaO_2_ + MnO_2_@PLGA MPs exhibited significantly reduced fluorescent intensity, suggesting that the hypoxic environment could be effectively reversed by the developed MPs. It has been reported that the probe Image-iT Green Hypoxia Reagent is fluorogenic when atmospheric oxygen levels are lower than 5% (Stone et al., 2019). Therefore, the absence of a hypoxia signal in the cells further implied that the local oxygen tension could be increased to higher than 5% by applying the CaO_2_ + MnO_2_@PLGA MPs developed in this study.

To further confirm the potential of CaO_2_ + MnO_2_@PLGA MPs in alleviating cellular hypoxia, the test cells were fixed and immunostained for HIF-1α. Under normally oxygenated conditions, the key hypoxia-responsive transcription factor HIF-1α is rapidly degraded in cytosol by prolyl hydroxylase (Maes et al., 2012; Min et al., 2015); when exposed to a low oxygen tension environment, HIF-1α begins to accumulate and translocate into nuclei to modulate downstream genes that may help relieve cellular hypoxia (Maes et al., 2012; Min et al., 2015). Therefore, both the amount of HIF-1α protein and its subcellular location can be employed as markers to evaluate cellular hypoxia. As shown in Figure 4C, under normoxic circumstances, no significant accumulation of HIF-1α was observed in cells. Following incubation under low oxygen tension (1% O_2_), the MC3T3-E1 cells exhibited enhanced nuclear HIF-1α staining. When the oxygen-generating CaO_2_ + MnO_2_@PLGA MPs were used, a significant proportion of the nuclear HIF-1α signal was eliminated in the cells that were exposed to low oxygen tension, suggesting that the developed CaO_2_ + MnO_2_@PLGA MPs may function as a local oxygen-evolving system effectively to relieve tissue hypoxia. According to the oxygen evolution profiles (Figures 1C, 2A) obtained using a dissolved oxygen sensor, the concentration of dissolved oxygen in the CaO_2_ + MnO_2_@PLGA MP-treated solution increased substantially, suggesting that the generated oxygen molecules can dissolve into aqueous solution. Hence, these dissolved oxygen molecules can diffuse freely in the culture medium and thus can be utilized by cells to relieve cellular hypoxia.

### CaO_2_ + MnO_2_@PLGA MPs Promote Osteogenic Differentiation of MC3T3-E1 Cells Under Hypoxia

It has been widely reported that low oxygen tension has a significantly deleterious effect on fractural heading and new bone formation through the inhibition of osteogenic differentiation of osteoblasts (Salim et al., 2004; Lu et al., 2013; Yang et al., 2013; Lin et al., 2014; Liu et al., 2019). To evaluate whether the developed MPs possess the potential to promote osteogenesis of hypoxic bone tissue, preosteoblast MC3T3-E1 cells that were incubated under low oxygen tension (1% O_2_) and treated with ascorbic acid and β-glycerophosphate to induce osteogenic differentiation and test MPs for improving local oxygenation were employed as an *in vitro* model. Calcium mineral deposition identified by Alizarin red staining was used as an index to assess the degree of *in vitro* mineralization and thus the osteogenic differentiation of MC3T3-E1 cells (Kuo et al., 2016). Cells that were cultivated under normal (20% O_2_) or low (1% O_2_) oxygen tension without MP treatment were used as controls.

**Figure 5:**
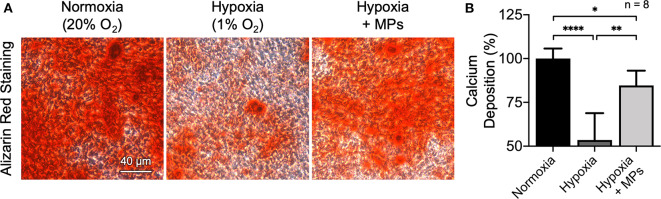
Enhanced osteogenesis of hypoxic MC3T3-E1 cells by CaO_2_ + MnO_2_@PLGA MPs. **(A)** Representative images of Alizarin red staining and **(B)** corresponding quantitative results showing calcium deposited by MC3T3-E1 cells following various treatments. *p < 0.05; **p < 0.01; ****p < 0.001.

According to the results of Alizarin red staining, more orange-red precipitate, namely higher calcium deposition, was observed in the cells cultivated under normal oxygenated conditions when compared to that in hypoxic culture (Figure 5A), suggesting that the low oxygen tension could apparently impair the osteogenic differentiation capacity of MC3T3-E1 cells. In the group that received test MPs, however, the osteogenic differentiation potential was restored significantly (Figure 5A). These results were further supported by the levels of calcium accumulation quantified by measuring the optical density at 562 nm (Figure 5B). Together with the previous results, these findings suggest that the developed CaO_2_ + MnO_2_@PLGA MPs can be utilized to enhance the oxygenation and thus *in vitro* osteogenic differentiation of preosteoblasts under low oxygen tension.

### The CaO_2_ + MnO_2_@PLGA MPs Are Biocompatible

**Figure 6:**
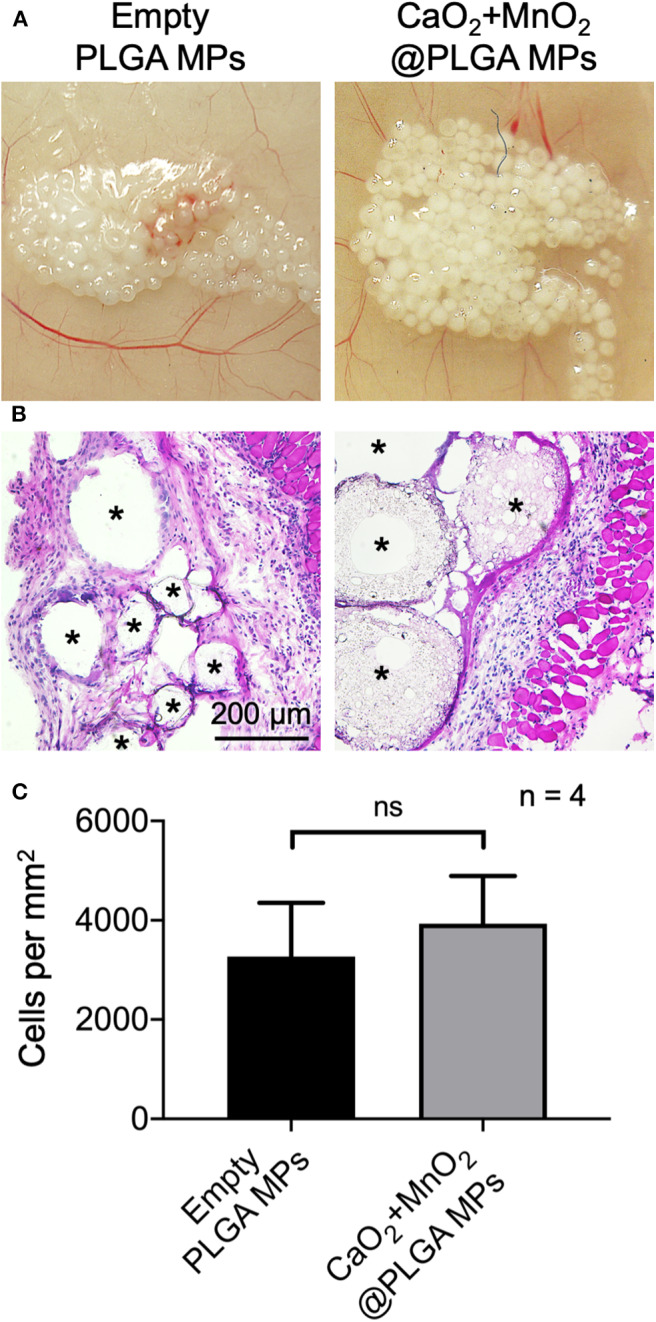
*In vivo* biocompatibility assessment of CaO_2_ + MnO_2_@PLGA MPs. Representative **(A)** photographs and **(B)** H&E staining images of mouse skin tissue after subcutaneous injection of test MPs and **(C)** corresponding quantification of infiltrating cells. Asterisks indicate the implanted MPs or the spaced originally occupied by MPs. ns, not significant.

The developed CaO_2_ + MnO_2_@PLGA MPs were implanted subcutaneously by local injection to evaluate their biocompatibility. Animals that received empty MPs that were composed of only PLGA, which is generally regarded as a biocompatible material and has been approved for clinical use by the US Food and Drug Administration (Gentile et al., 2014), were used as controls. At 2 weeks after implantation, the MPs in both groups were integrated into the subcutaneous tissue without inducing macroscopic inflammation (Figure 6A). The images of H&E staining (Figure 6B) and the corresponding results of cell nuclei counting (Figure 6C) revealed that the CaO_2_ + MnO_2_@PLGA MPs exhibited a similar trend of host cell infiltration when compared to the control group (*p* > 0.05), suggesting that the encapsulation of 10 mg/mL CaO_2_ and 0.22 mg/mL MnO_2_ did not result in significant alternation of PLGA MP biocompatibility. Therefore, we speculate that the developed CaO_2_ + MnO_2_@PLGA MPs can be biocompatible as empty PLGA MPs.

## Conclusion

This study pursued to elucidate the relationship among the concentrations of each component used for CaO_2_ + MnO_2_@PLGA MP fabrication, the resultant oxygen release behaviors, and their corresponding cytotoxicity for the purpose of engineering an optimal injectable oxygen-generating carrier. A series of methodologies were used to evaluate the impacts of these parameters, and the obtained results evidently demonstrate that all the components of the MPs play crucial roles in modulating the oxygen release behavior and cytotoxicity, as measured by the dissolved oxygen concentration, pH value, H_2_O_2_ concentration, and cell viability. The resultant CaO_2_ + MnO_2_@PLGA MPs developed in this study could effectively relieve the hypoxia of MC3T3-E1 cells that were grown under low oxygen tension and promote their osteogenic differentiation, thus holding great promise in enhancing fractural healing by increasing tissue oxygenation.

## Data Availability Statement

The datasets generated for this study are available on request to the corresponding author.

## Ethics Statement

This animal study was reviewed and approved by Institutional Animal Care and Use Committee, National Tsing Hua University.

## Author Contributions

T-EH, P-LL, and C-CH contributed to conception and design of the study. T-EH, S-JL, L-CC, and C-CC carried out the experimental work. T-EH, S-JL, L-CC, and C-CH perform the data analysis. P-LL and C-CH supervised the research design and wrote the manuscript. All authors approved the final version of the manuscript.

## Conflict of Interest

The authors declare that the research was conducted in the absence of any commercial or financial relationships that could be construed as a potential conflict of interest.
