# Supplementary material for: Optimizing an Injectable Composite Oxygen-Generating System for Relieving Tissue Hypoxia
Source: Front Bioeng Biotechnol. 2020 May 26;8:511. doi: 10.3389/fbioe.2020.00511 (PMC7264163; doi:10.3389/fbioe.2020.00511)
Supplement: Supplementary file 1 [file Data_Sheet_1.docx]

Supplementary Material

## Supplementary Figure


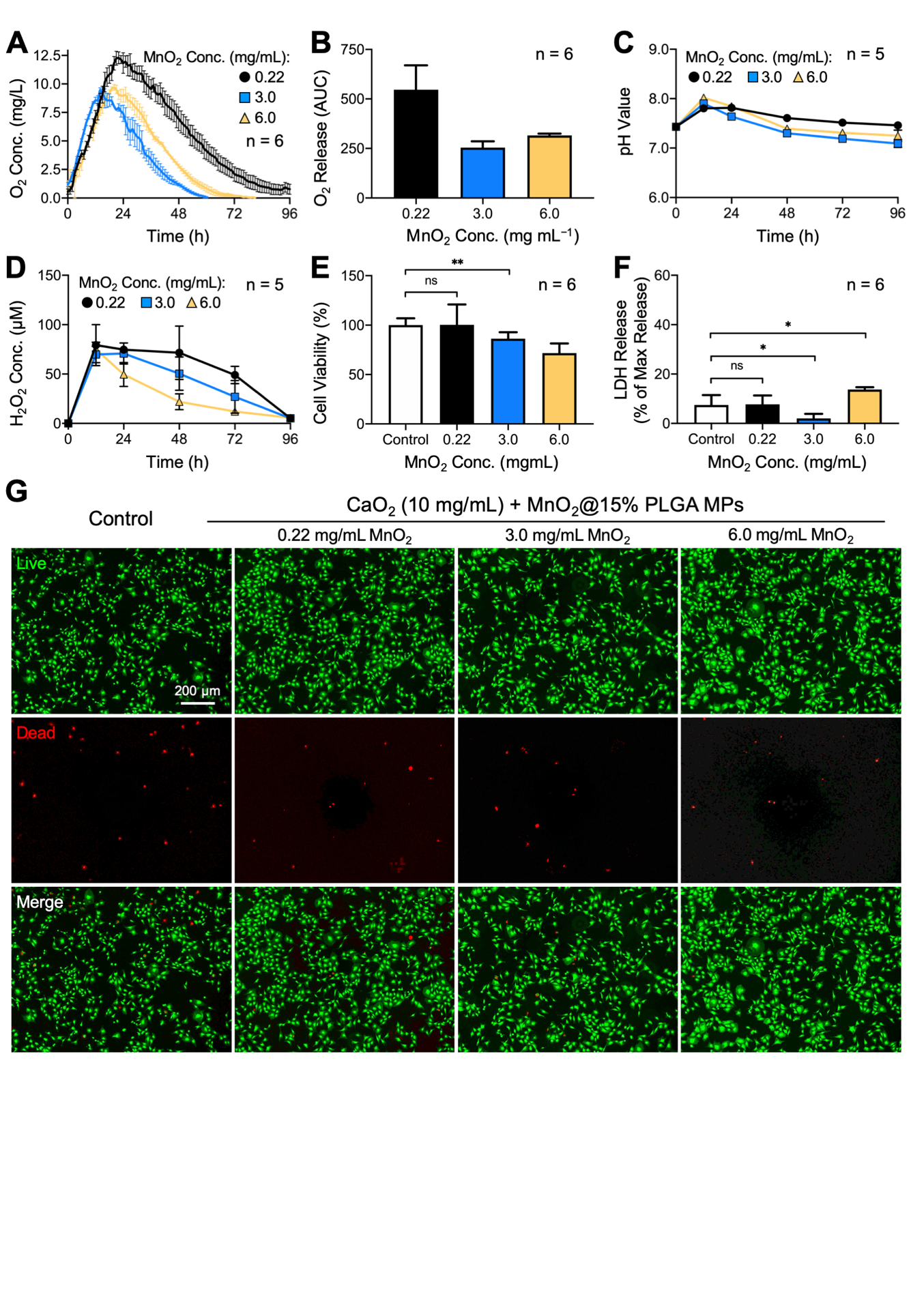


**Supplementary Figure 1.** Effects of the amount of encapsulated MnO_2_ on the oxygen release behavior of CaO_2_ + MnO_2_@PLGA MPs. (a) The release profiles and (b) corresponding AUCs of oxygen release from MPs. (c) Variation in pH and (d) accumulation of H_2_O_2_ in PBS samples following treatment with MPs. (e) Results of the CCK-8 assay, (f) LDH assay, and (g) live/dead staining following treatment with MPs. **p* < 0.05; ***p* < 0.01; ns, not significant.


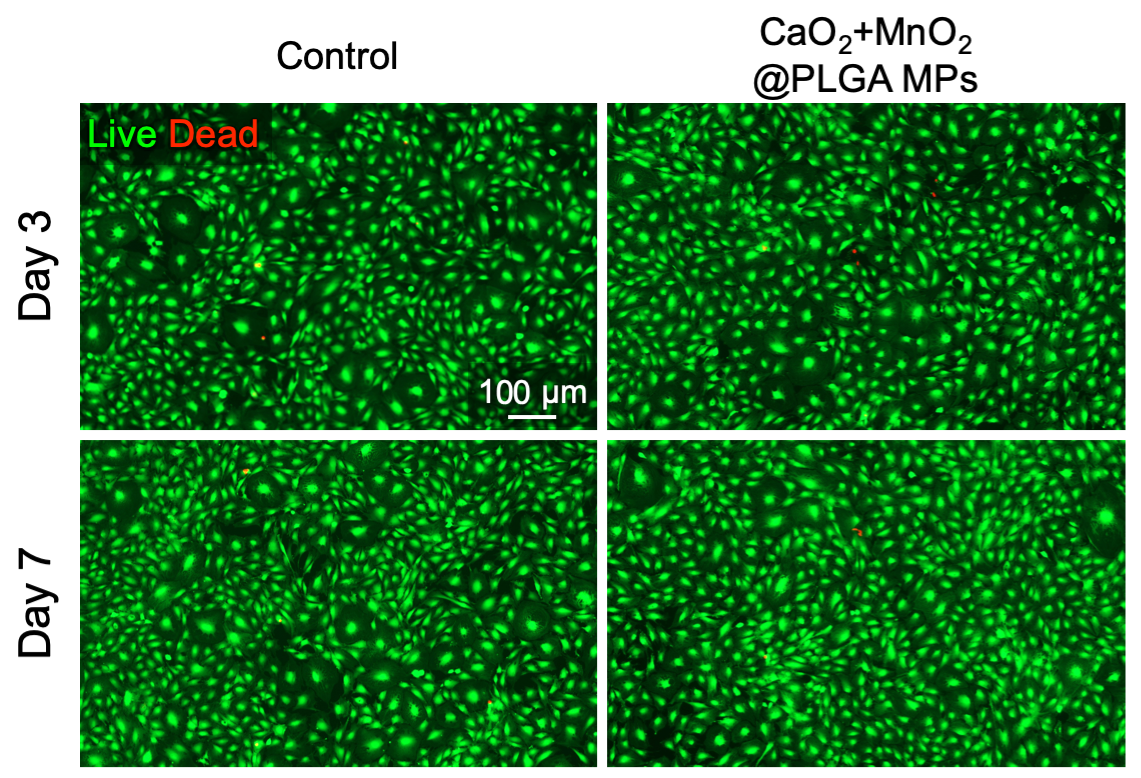


**Supplementary Figure 2.** Representative live/dead images of the MC3T3-E1 cells receiving the developed CaO_2_ + MnO_2_@PLGA MPs for 3 or 7 days.
